# Harnessing genetic potential of wheat germplasm banks through impact-oriented-prebreeding for future food and nutritional security

**DOI:** 10.1038/s41598-018-30667-4

**Published:** 2018-08-21

**Authors:** Sukhwinder Singh, Prashant Vikram, Deepmala Sehgal, Juan Burgueño, Achla Sharma, Sanjay K. Singh, Carolina P. Sansaloni, Ryan Joynson, Thomas Brabbs, Cynthia Ortiz, Ernesto Solis-Moya, Velu Govindan, Naveen Gupta, Harminder S. Sidhu, Ashwani K. Basandrai, Daisy Basandrai, Lourdes Ledesma-Ramires, Maria P. Suaste-Franco, Guillermo Fuentes-Dávila, Javier I. Moreno, Kai Sonder, Vaibhav K. Singh, Sanjay Singh, Sajid Shokat, Mian A. R. Arif, Khalil A. Laghari, Puja Srivastava, Sridhar Bhavani, Satish Kumar, Dharam Pal, Jai P. Jaiswal, Uttam Kumar, Harinder K. Chaudhary, Jose Crossa, Thomas S. Payne, Muhammad Imtiaz, Virinder S. Sohu, Gyanendra P. Singh, Navtej S. Bains, Anthony Hall, Kevin V. Pixley

**Affiliations:** 1 International Maize and Wheat Improvement Center (CIMMYT), Carretera México-Veracruz Km. 45, El Batán, Texcoco, C.P. 56237 Mexico; 2 Department Plant Breeding & Genetics, Punjab Agriculture University, Ludhiana, 141004 India; 3 ICAR-Indian Institute of Wheat and Barley Research, Karnal, 132001 India; 4 Earlham Institute, Norwich, Norfolk NR4 7UG UK; 5 Carretera Celaya-San Miguel de Allende, Km 0.6.5, C.P., 38110 Celaya, Guanajuato Mexico; 6 Borlaug Institute for South Asia (BISA), CIMMYT, Ladhowal, Punjab, 141004 India; 7 CSK Himachal Pradesh Agricultural University Palampur, Palampur, Himachal Pradesh 176062 India; 8 INIFAP-CIRNO, Campo Experimental Norman E. Borlaug, Apdo. Postal 155, Km 12 Norman E. Borlaug, Cd. Obregon, Sonora, C.P. 85000 Mexico; 9 INIFAP, Interior Parque Los Colomos S/N, Colonia Providencia, CP, 44660 Guadalajara, Jalisco Mexico; 10 ICAR-Indian Agricultural Research Institute (IARI), New Delhi, 110 012 India; 11 National Research Center for Plant Biotechnology, New Delhi, 110 012 India; 12 Nuclear Institute for Agriculture and Biology, Faislabad, 38000 Pakistan; 13 Department of Plant and Environmental Sciences, Crop Science, University of Copenhagen, Højbakkegård Allé 13, DK-2630 Taastrup, Denmark; 14 Nuclear Institute of Agriculture, Tando Jam, Sindh 70050 Pakistan; 15 CIMMYT - World Agroforestry Centre (ICRAF), United Nations Avenue, Gigiri. P.O. Box 1041–00621, Nairobi, Kenya; 16 ICAR-Indian Agricultural Research Institute, Regional Station, Shimla, 171004 India; 17 Department of Genetics and Plant Breeding, G.B. Pant University of Agriculture & Technology, Pantnagar, 263145 Uttarakhand India; 18 CIMMYT – Pakistan, NARC CSI Complex, Park Road, Islamabad, 44000 Pakistan; 19 School of Biological Sciences, University of East Anglia, Norwich Research Park, Norwich, NR4 7TJ UK

## Abstract

The value of exotic wheat genetic resources for accelerating grain yield gains is largely unproven and unrealized. We used next-generation sequencing, together with multi-environment phenotyping, to study the contribution of exotic genomes to 984 three-way-cross-derived (exotic/elite1//elite2) pre-breeding lines (PBLs). Genomic characterization of these lines with haplotype map-based and SNP marker approaches revealed exotic specific imprints of 16.1 to 25.1%, which compares to theoretical expectation of 25%. A rare and favorable haplotype (GT) with 0.4% frequency in gene bank identified on chromosome 6D minimized grain yield (GY) loss under heat stress without GY penalty under irrigated conditions. More specifically, the ‘T’ allele of the haplotype GT originated in *Aegilops tauschii* and was absent in all elite lines used in study. *In silico* analysis of the SNP showed hits with a candidate gene coding for *isoflavone reductase* IRL-like protein in *Ae. tauschii*. Rare haplotypes were also identified on chromosomes 1A, 6A and 2B effective against abiotic/biotic stresses. Results demonstrate positive contributions of exotic germplasm to PBLs derived from crosses of exotics with CIMMYT’s best elite lines. This is a major impact-oriented pre-breeding effort at CIMMYT, resulting in large-scale development of PBLs for deployment in breeding programs addressing food security under climate change scenarios.

## Introduction

The twentieth century witnessed the impact of traditional breeding methods in enhancing genetic gains for wheat grain yield (GY)^1^. In the pre-Green Revolution period, breeding efforts relied on selection from locally adapted landraces or traditional cultivars, but a paradigm shift occurred with the extensive use of semi-dwarf wheat cultivars beginning during the Green Revolution. Further, the development and use of synthetic wheats in breeding brought diverse alleles from the tertiary gene pool into varietal pipelines^2,3^. These and other advances in breeding enabled annual genetic gains for GY between 0.6 and 1.0 percent for varieties released between 1994 and 2010^4^. However, rates of gain must increase to meet the food and nutrition demand of our growing global population under the likely, unfavorable climate change scenarios.

The impact of landraces, wild relatives and synthetic wheats on genetic gains for GY, by providing resistance against biotic stresses, and to a much lesser extent against abiotic stresses, is well recognized^5–8^. Nevertheless, breeders are generally reluctant to use exotic genetic resources because of the long-term commitment required to identify useful, novel diversity and introgress it into well-adapted elite cultivars while reducing the effects of undesired, linked genes. A commonly reported, intriguing analogy, is that the “bad” parent of good × bad crosses often contributes one or more QTL with favorable effects on the trait of interest^9^. Approximately 800,000 wheat accessions, including landraces and synthetics, exist in germplasm banks globally as reservoirs of useful alleles for breeding, but only a very small proportion of these have been used in varietal improvement programs^10^. Low-cost high-density genomics and growing bioinformatics capacity are increasing the feasibility of identifying and using the “diamonds in the rough” within these genetic resources.

**Figure 1:**
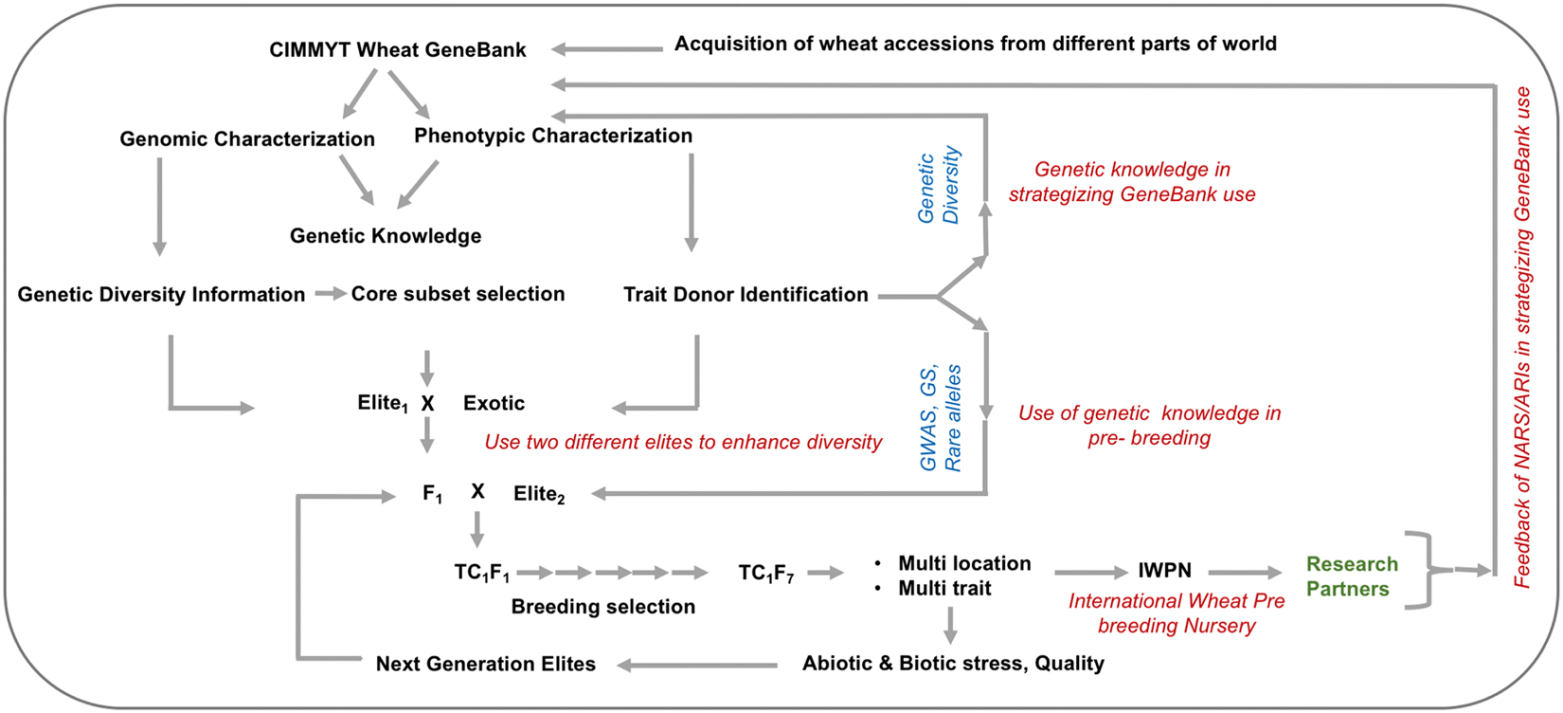
Proposed and reported wheat pre-breeding schemes. Germplasm bank accessions are genotyped while field and laboratory phenotyping is performed for various traits using sub-sets or core sub-sets of accessions. Genotypic and phenotypic information are used to form core subsets for phenotyping. Once trait donors are identified, these are used for crossing with elite lines (exotic/elite1//elite2), followed by selection under heat, drought and disease conditions during TC_1_F_2_ to TC_1_F_5_ generations. The advanced genotypes are distributed (these are currently available) on request to researchers across the world.

Pre-breeding, or the development of “half-way” germplasm introgressing exotic (unimproved) into elite germplasm, plays a key role in broadening the genetic base of breeding germplasm pools. Pre-breeding programs that create freely available, pre-competitive, bridging or intermediate germplasm by introgressing desirable genes from exotic into elite lines empower breeding programs to use germplasm bank diversity cost effectively. Realizing this, a few wheat pre-breeding projects have been launched^11,12^. CIMMYT’s Seeds of Discovery^13^ is an innovative project initiated to use next generation genome sequencing technology, envirotyping and analytical methods to characterize and enhance the use of germplasm bank accessions^14–16^. This is the third major germplasm infusion effort at CIMMYT that has resulted in strategic development of large-scale pre-breeding germplasm (Fig. 1) followed by its deployment through breeding pipelines. Useful and novel diversity for stress tolerance (heat, drought, yellow rust and powdery mildew) and quality (zinc concentration) was introgressed to lines derived from crosses of exotics with CIMMYT’s best elite wheat germplasm. Concomitantly, this research identified rare haplotypes effective against abiotic or biotic stresses contributed from exotics through haplotypes-based genome wide association study.

## Results

### Analysis of Exotic Genome in Pre-Breeding Lines (PBLs)

**Figure 2:**
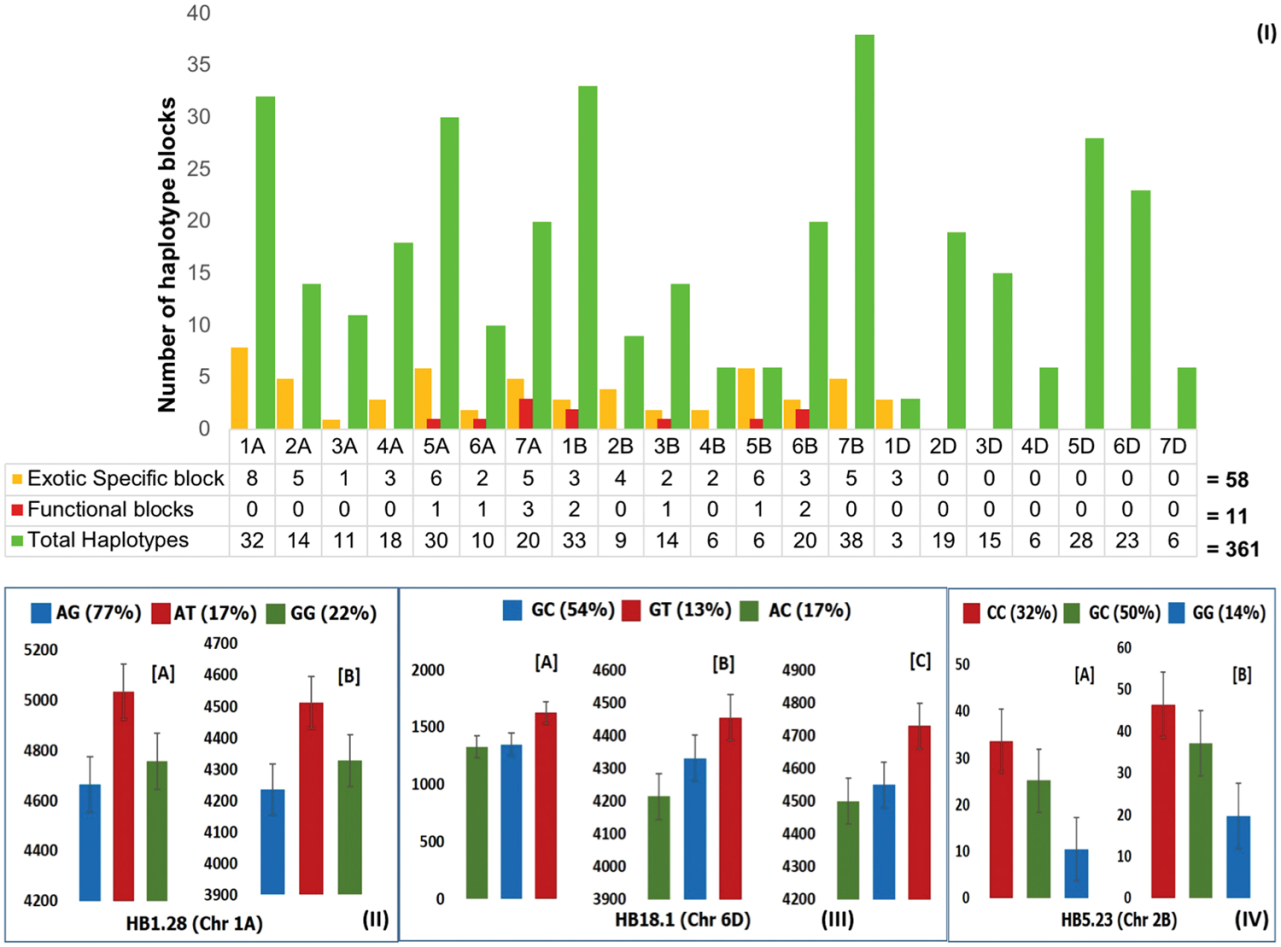
(**I**) Total number of haplotype blocks (HBs), number of HBs introgressed from exotic parents, and number of functional exotic-specific HBs (associated with traits investigated in the study: diseases, heat, drought etc.) on each chromosome for the 984 pre-breeding lines (PBLs). (**II**) Average grain yield (Kg/ha) and frequency (%) of PBLs for each haplotype, AT, AG, and GG of HB1.28 grown under irrigation [A] or drought stress [B] at Ciudad Obregon, Mexico and Karnal, India, respectively. Y-axis = grain yield, X-axis = haplotype classes. (**III**) Average grain yield (Kg/ha) and frequency (%) of PBLs with each haplotype, GT, GC and AC of HB18.1 grown under heat stress at Ciudad Obregon, Mexico [A], and drought [B], and irrigated [C] conditions at Karnal, India. Y-axis = grain yield; X-axis = haplotype classes. (**IV**) Mean yellow rust disease severity and frequency (%) of PBLs with haplotypes CC, GC and GG of HB5.23. The PBLs were evaluated in Ludhiana, India during 2015 [A] and 2016 [B]. Y-axis = disease severity (%), X-axis = haplotype classes. (**II**–**IV**) are for evaluation of a sub-set of 134 of the 984 PBLs.

Haplotype block analysis for the complete set of 984 PBLs, performed by localizing the genome-wide SNPs to a high-density consensus map (available at http://www.diversityarrays.com/sequence-maps), resulted in 361, 115 and 367 haplotype blocks (HBs) in pre-breeding lines (PBLs), elite and exotic parents, respectively, based on average linkage disequilibrium (LD) distance of 5 cM. Supplementary Table 1 describes haplotype variation among PBLs, exotic and elite parental lines. There were fewer and larger HBs in elite compared to exotic parents and PBLs on all chromosomes except 6D and 7D. For example, a series of 36 SNPs on chromosome 1A were grouped in one very large HB (~67.3 cM) in the parental elite lines, these SNPs were distributed into 9 HBs (2–10 cM, 2–6 SNPs) in the PBLs (Supplementary Fig. 1**)**. Haplotype block-by-block comparison by chromosome revealed that 58 (16%) of the 361 HBs identified in PBLs originated from or were specific to their exotic parents. Of the 58 exotic-specific HBs in PBLs, 11 (19%) were positively associated with agronomic traits and disease resistance [Fig. 2(I)**]**. Elite-specific HBs were not estimated in PBLs due to the small number of elite parents used.

### SNP Allele Frequency Analysis for PBLs from 10 Crosses

In another approach to investigate the exotic parent contribution to PBLs, SNP allele frequencies were evaluated for 278 PBLs derived from 10 crosses, each with progeny number ≥16, involving 9 different exotic and 7 elite parents. Of the homozygous PBL alleles that could be traced to either exotic or elite parents, an average of 23.4% were inherited from their exotic parents and 65.9% from one of their elite parents. Exotic introgression patterns varied among chromosomes (Supplementary Table 2, Supplementary Fig. 2). Most of the SNP alleles in the PBLs were present in both their respective exotic and elite parents; however, of the 24.5% (11.3–47.4%) for which parental origin could be determined, 25.1% (18.9–33.9%) originated from the exotic parent, 70.8% from one or both elite parents and 4.1% were heterozygous. If we include 6.9% missing markers in these calculations, an average of 23.4% of SNP markers were inherited from their exotic parent, 65.9% from elite parents and 3.8% were heterozygous. These frequencies correspond closely with expectations of 25% exotic and 75% elite alleles for a TC_1_F_5_ (top cross) population. Supplementary Fig. 2 illustrates exotic- and elite-specific imprints in genomes of PBLs in the analyzed crosses. Chromosome 2A, for example, had a region where alleles appeared to be preferentially inherited from the elite parents, while chromosomes 3B and 5B had segments where alleles from exotic parents prevailed in the PBLs. It will be interesting to study these genomic regions in depth to increase our understanding of preferential accumulation of exotic and elite specific alleles in these PBLs.

### Haplotype-Trait Associations

The genome wide association (GWA) analysis identified HBs significantly associated with grain yield (Table 1 and Supplementary Tables 3 and 4) and disease resistances under multiple environments (Supplementary Table 5A,B). Among HBs that had significant effects on grain yield and biomass across multiple drought, heat and irrigated sites, HBs 10.5, 18.1 and 19.24 were of particular interest because they had significant effects at 7 to 11 of the 20 trait evaluation instances, and were not associated with days to heading (Supplementary Table 3). On the other hand, HB17.5 also had multiple instances of significant association with grain yield and biomass, but was also associated with days to heading, suggesting that maturity may have contributed to escaping heat or drought stress. Three HBs, HB16.10, HB18.2 and HB19.3 were associated with grain yield under heat stress, but also with days to heading (Supplementary Table 3).

**Table 1:** Consistent genomic regions (haplotype blocks) for grain yield and related traits.

Haplotype	Chr	984–2016	134–2016	134–2017	134–2017	984–2016	984–2017	134–2016	984–2016	134–2016	134–2017	134–2017	134–2017	134–2017	984–2016	984–2017	134–2016	134–2017	134–2017	984–2016	984–2016	984–2016	984–2016
		Grain yield (Kg/ha)													Biomass					Days to heading			
		Obre-gon-DRT	Obre-gon-DRT	Obre-gon-DRT	IIWBR-DRT	Obre-gon-HT	Obre-gon-HT	Obre-gon-HT	Obre-gon-IRGT	Obre-gon-IRGT	Obre-gon-IRGT	BISA-IRGT	IIWBR-IRGT	PAU-IRGT	Obre-gon- DRT	Obre-gon-HT	Obre-gon-DRT	IIWBR-DRT	IIWBR-IRGT	Batan-IRGT	Obre-gon-IRGT	Obre-gon DRT	Obre-gon HT
HB1.28	1A				**				**	**								**					
HB2.13 (Exotic HB)	1B	**								**													
HB4.1	2A				**	**						**	**		**			**	**				
HB4.13	2A				**								**			**			**				
HB5.3	2B					**								**				**		**		**	
HB10.5	4A	**			**	**			**			**	**					**	**				
HB10.7	4A					**			**			**									**		
HB14.31	5B						**						**			**		**	**				
HB16.10	6A					**	**		**							**					**	**	
HB17.1 (Exotic HB)	6B						**	**	**														
HB17.5	6B	**				**			**					**		**		**			**		**
HB17.6	6B			**				**	**			**	**								**		
HB18.1	6D				**	**	**	**	**				**		**	**	**	**	**				
HB18.2	6D					**	**		**							**					**		**
HB19.3 (Exotic HB)	7A					**		**	**												**		
HB19.24	7A				**	**	**					**	**		**				**				

**P ≤ 0.01; Chr: Chromosome, DRT: Drought, HT: Heat, IRGT: Irrigated; Obregon: Ciudad Obregon, Mexico, BISA: Borlaug Institute of South Asia, India, PAU: Punjab Agriculture University, India, IIWBR: Indian Institute of Wheat and Barley Research, India.

Two pre-breeding germplasm populations of 984 and 134 accessions evaluated across the locations in crop seasons of 2016 and 2017. The 134 accessions were part of 984 set.

On chromosome 2B, a rare haplotype of HB5.23, GG (present in 14% of PBLs), was associated with yellow rust (*Puccinia striiformis* f. sp. *tritici*) resistance in both years of evaluation [Fig. 2(IV)]. For powdery mildew (*Blumeria graminis* f. sp*. tritici – Bgt*), two genomic regions on 5B and 6B had significant effects (Supplementary Table 5B). A rare haplotype of HB14.36 (5B) was identified in 6.7% of PBLs, and 90% of the lines with this haplotype were resistant or moderately resistant to powdery mildew. Similarly, a rare haplotype of HB17.11 (6B) was identified in 10.5% of PBLs, and 83% of the lines with this haplotype were resistant or moderately resistant to powdery mildew.

### Characterization of Rare Haplotypes

**Figure 3:**
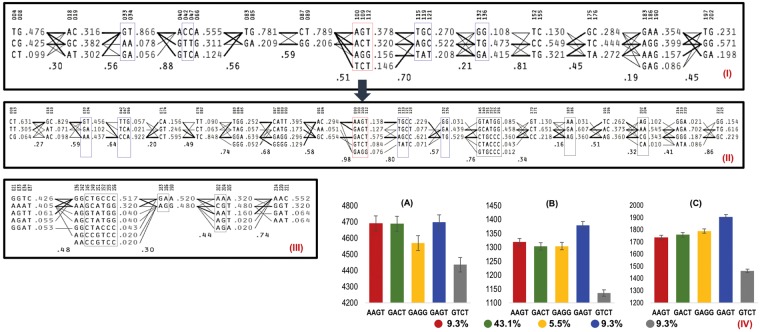
Haplotype block (HB) map of chromosome 6A in exotic parents (**I**), pre-breeding lines (PBLs) (**II**) and elite parents (**III**). Each haplotype is displayed in a HB with its population frequency indicated on the right. The value shown below and between HBs represents multi-allelic D’, which indicates the level of recombination between the two blocks. HBs partly enclosed in blue or black indicate introgressions from exotic or elite parents into the PBLs, respectively. HBs enclosed in red are from exotic and had significant effects for the trait. (**IV**) Allelic effects of HB16.10 in the PBLs: haplotype GAGT produced grain yield advantage under heat-stress in 2015 [IV-B] and 2016 [IV-C], with no disadvantage under irrigated conditions [IV-A] at Ciudad Obregon, Mexico. Haplotypes and their frequency (%) among 984 PBLs are plotted on the X-axis.

Figure 2II–IV shows the positive effects of rare haplotypes of HB1.28 (chromosome 1A) and HB18.1 (chromosome 6D) on GY and of HB5.23 (chromosome 2B) on yellow rust resistance. The SNP alleles of the rare haplotype of HB5.23 were derived from exotic parents. For powdery mildew, two rare haplotypes on chromosomes 5B (from exotic parent) and 6B (from elite parent) had significant effects (Supplementary Table 5B). Figure 3 presents the effect of rare haplotype of HB16.10 (6A) on GY under heat stress.

The HB5.23, located on chromosome 2B and associated with yellow rust resistance has a size of ~32 Mbp and contains 279 high confidence genes including 10 with nucleotide-binding and leucine-rich repeat (NB-LRR) domains known to interact with pathogen effectors to induce defense responses. Of the GY-associated HBs; HB10.5 spanned ~13.8 Mbp containing 61 genes, HB16.10 spanned ~28.5 Mbp containing 138 genes and HB18.1 spanned ~2.3 Mbp containing 48 genes. Using Knetminer^17^, we identified 4, 14 and 6 candidate genes (Supplementary Table 6) for haplotypes HB10.5, HB16.10 and HB18.1, respectively.

**Figure 4:**
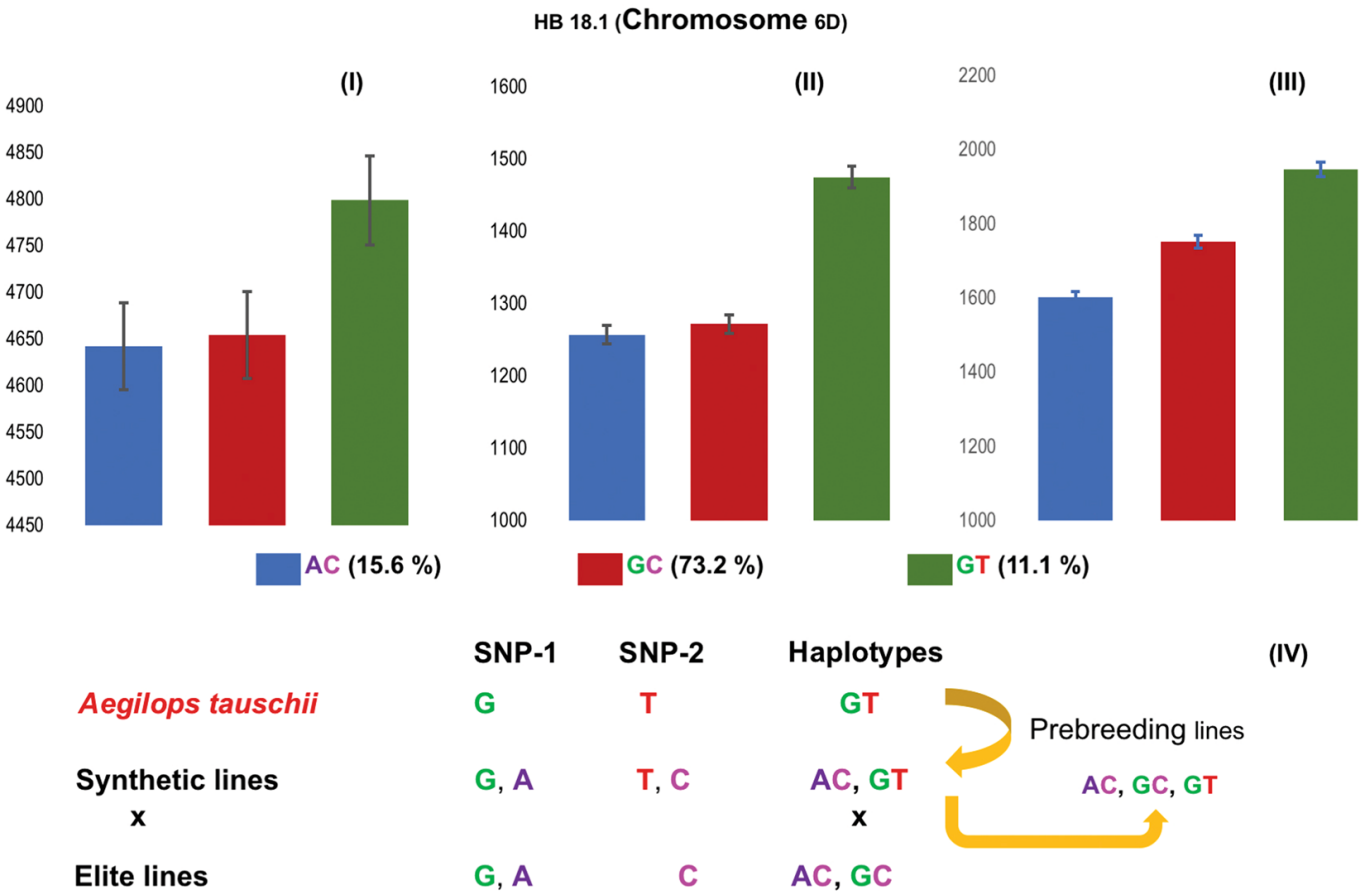
Average grain yield (Y-axis) and frequency (%) of PBLs with alleles AC, GC and GT (X-axis) for HB18.1. PBLs with haplotype GT had highest grain yield across 2016 irrigated (A-**I**), 2016 heat-stress (A-**II**); and 2017 heat-stress (A-**III**) at Ciudad Obregon, Mexico. (A-**IV**): The origin of the yield-increasing haplotype allele, GT, was from synthetics that acquired the ‘T’ SNP allele from *Aegilops tauschii*. The favorable GT haplotype was present in *Ae. tauschii*, synthetics and PBLs, and was absent in elite parents of the 984 PBLs.

Figure 4 shows that the favorable and rare haplotype GT of the block HB18.1, associated with grain yield advantage under heat stress, inherits the ‘T’ allele from *Aegilops tauschii* via synthetic pedigree. Further, this SNP (belonging to clone ID 1067078) showed similarity with a candidate gene Traes_6DS_84A4D85F.1 through BLAST analysis. This gene is homologous to a rice gene LOC_Os06g27770.1 coding for isoflavone reductase. Phylogenetic analysis revealed a high level of similarity of Traes_6DS_84A4D85F.1 with the gene F775_22033 in *Ae. tauschii*, also coding for isoflavone reductase IRL-like protein (Supplementary Fig. 3). Analysis of allelic variants of this gene showed eight missense mutations (causing deleterious amino acid changes) with SIFT score <0.05 in the coding region (Supplementary Fig. 4), of which seven were SNPs and one was a 2 bp substitution (Supplementary Table 7). In rice, isoflavone reductase-like gene (*OsIRL*) has been shown to be involved in homoeostasis of reactive oxygen species^18^. In wheat, detailed physiological dissection of this gene is underway to identify the underlying mechanism conferring heat tolerance.

### Pre-Breeding Lines for Use as Trait Donors

Supplementary Table 8A,B provide grain yield data for the highest-yielding PBLs and elite checks grown under heat and drought stress conditions during two seasons. Five PBLs originating from five crosses had GY at par with the best checks Baj#1, and Vorobey under drought stress (Supplementary Tables 8B). However, none of the PBLs significantly out-yielded the tolerant checks in two years consecutively. This points to high genetic potential of checks as compared to PBLs. To dissect the genomic regions providing high yield advantage under drought and heat stresses particularly in Baj#1, we have initiated genetic dissection studies. Preliminary analysis has identified a genomic region on 4A, which is specific to Baj#1 and Baj#1-derived lines (results not shown).

**Figure 5:**
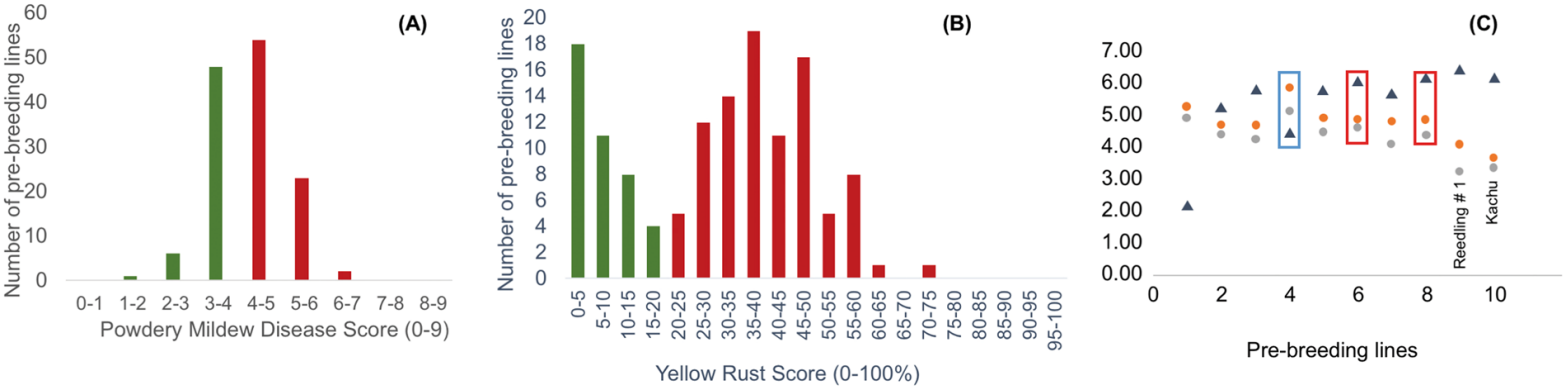
(**A**) Powdery mildew disease symptom scores (0 = resistant to 9 = susceptible) and (**B**) Yellow rust severity (%) for 134 pre-breeding lines PBLs. Green and red bars indicate resistant and susceptible PBLs, respectively. (**C**) Y-axis, grain yield (Mg/ha, black triangles) and zinc content [(µg g^−1^ × 0.1), orange (2015) and grey (2016) dots] for 8 PBLs and 2 check lines (X-axis). PBLs 6 and 8 had similar grain yield but significantly higher zinc content as the checks, whereas PBL 4 had very high zinc content (up to 18 µg g^−1^ higher) but low grain yield. LSD for zinc content in 2015 and 2016 was 6.8 and 6.3 µg g^−1^, respectively.

PBLs with resistance to yellow rust and powdery mildew are shown in Fig. 5A,B. Thirteen PBLs had yellow rust symptom score ≤5% (0 and 100% being completely resistant and susceptible, respectively), and six PBLs had powdery mildew symptom scores ≤2.5 on a 0–9 scale (0 and 9 being completely resistant and susceptible, respectively).

Six PBLs had greater (p < 0.05) Zn concentration than the best check line, Reedling#1 in the two years’ evaluation. One PBL (GID: 7640819, CROC_1/AE.SQUARROSA (481)//KACHU/3/BAJ #1) had 18 and 17 µg g^−1^ more Zn than the check in years 1 and 2, respectively (Fig. 5C). The Pearson’s correlation of Zn concentration in grain with grain yield was r = −0.21 (p < 0.05), which was consistent with previous reports of negative association between these traits^19^.

## Discussion

Directional selection, either natural or through breeding, increases the frequency of favorable alleles resulting in the formation of conserved haplotypes with strong surrounding linkage disequilibrium^20^. Wheat has been exposed to intense artificial (through breeding) and natural selection^21^ since its domestication, resulting in large HBs as observed for the elite germplasm evaluated herein. These HBs may inadvertently fix unfavorable alleles linked with selected genes; for example, as demonstrated by Voss-Fels *et al*.^22^ for root traits negatively affected by linkage drag with the selected *Vrn* gene for heading date. Thus, HBs prevalent in elite germplasm may need to be broken to introduce and capture valuable diversity within them that may otherwise remain undiscovered and unused. The pre-breeding strategy reported here successfully disrupted many large HBs present in the elite lines (e.g. Supplementary Fig. 1). Bevan *et al*.^23^ have described how the assembly of rare, favorable haplotypes, such as those identified herein, may contribute to near-future breeding strategies.

As per Mendelian genetics, in a three-way cross of exotic with two elite parents, genomic contribution of exotic is expected to be approximately 25%. To quantify exotic contribution here, haplotypes maps of parental exotics and PBLs were compared and two independent calculations were made: first, using 156 PBLs and 156 exotics, and second, using all 984 PBLs and all 244 exotics. The genomic contribution of exotics in the first and second method was 15.2% (data not shown) and 16.1% [Fig. 2(I)], respectively. The first analysis was done to keep same size of the exotic and PBL populations, thereby eliminating possible confounding effect due to sample size differences. The fact that these estimates are below the theoretical 25% is not unexpected because these estimates are confounded and reduced by any HBs that may have been present in both exotic and elite parents. A third and traditional approach, wherein frequencies of exotic- and elite-specific SNP alleles were determined in 10 selected crosses (like in bi-parental populations), estimated the exotic genome contribution to PBLs very close to the theoretical 25%.

### Discovering Rare Haplotypes

The identification of rare haplotypes in HBs on chromosomes 6A and 6D associated with GY across environments, and on chromosome 2B for yellow rust resistance demonstrated the value of crosses with exotic germplasm (Figs 2 and 3). The GY advantage associated with favorable alleles in HBs 8.22, 18.1, and 19.24 may be due to increased biomass as these showed positive associations with both biomass and GY (Supplementary Table 3). Detailed dissection of these HBs revealed that HBs 8.22 and 19.24 increased biomass without affecting harvest index, and hence might have positive effect on other yield component(s). A closer HB to HB18.1 on chromosome 6D i.e. HB 18.2 (within 5 cM of HB18.1) showed association with thousand kernel weight (TKW) and a minor haplotype AC (present in 6% of PBLs) was favorable resulting in an average TKW of 47.6 g (4.5 to 6% more TKW than remaining two haplotypes; Supplementary Fig. 5). Thus, HB18.1 seems to increase GY via increase in both biomass and TKW. These HBs did not show any association with days to heading (independent of confounding effects of days to heading), they may be very useful for breeding programs.

It is noteworthy that the rare haplotype (GT) of HB18.1, which had a significant positive effect on biomass and grain yield of PBLs under drought and heat stresses, was inherited from *Ae. tauschii* via synthetic wheat (*Ae. tauschii* × *Triticum durum*) parents. More specifically, the ‘T’ allele of haplotype GT was absent in all elite parents studied here, whereas it was present in the exotic (synthetic) parents and their derived PBLs. Following this discovery, we screened 62,000 previously sequenced CIMMYT germplasm bank accessions for the presence of this favorable haplotype and found it in only 262 (0.42%) accessions. The majority of germplasm bank accessions with the ‘T’ allele were synthetic-derived lines such as Sokoll (released in 1997). All of the PBLs with the ‘T’ allele was susceptible to stem rust (*Puccinia graminis* f. sp. *tritici*), which may be coincidental or could suggest that selection for stem rust resistance led to negative selection of this haplotype. This would be similar to the inadvertent selection for the *Lr67* susceptible allele, which was associated with intense selection for *RhtD1b* semi-dwarf gene in CIMMYT’s elite germplasm^24^.

To follow-up on the candidate gene within HB18.1 (that affected grain yield and biomass under heat and drought stress) (Fig. 4), we have converted seven missense mutations with a SIFT score <0.05 to KASP assays. Future work will test whether natural variations in the gene are associated with grain yield and yield components under heat stress conditions. Supplementary Table 9 lists rare haplotypes inherited from exotics that are associated with traits investigated in the study and are useful for trait improvement programs.

The rare and favorable haplotype of HB5.23 for yellow rust resistance had a frequency of 19% in 62,000 germplasm bank accessions investigated. Ten NBS-LRR genes, which are well-known disease resistance proteins in plants^25^, were identified within the HB5.23 intervals. Most of these genes were located in a small cluster, similar to those observed in Arabidopsis^26^ and rice^27^, with 5 located within a 115 Kbp region and 3 within a sub-cluster of 22 Kbp. Nine genes viz. *Yr5*, *Yr7*, *Yr27*, *Yr31*, *YrV23*, *YrSp*, *YrQz*, *YrTp1*, and *YrCN19*^28^ showing resistance to multiple yellow rust strains have previously been mapped on chromosome 2B. Most of these known yellow rust genes, except *Yr5*, are not effective under Punjab conditions, where the association reported herein was detected. Molecular marker analysis confirmed that HB5.23 is not linked with *Yr5* (data not shown), and warrants an in-depth analysis to determine the novelty of the identified gene.

### Trait Value of Pre-Breeding Germplasm

Incorporation of new variation into elite materials broadens the genetic base and results in novel allele combinations for evaluation and selection in breeding programs. The PBLs described herein harbored gene(s) or allele(s) for disease resistance (Supplementary Tables 5A,B**)**, for GY advantage under heat and drought (Supplementary Tables 8A,B), and for high zinc concentration (Fig. 5C). Our results indicate that the exotic parents contributed useful diversity for prioritized (drought and heat tolerance) and un-prioritized (zinc content) traits. The baseline grain Zn content among commercial varieties is generally 25 ppm, and +12 ppm is the breeding target to enhance grain Zn in elite wheat lines to have nutritional impact. We identified lines with much higher Zn content than checks (Fig. 5C). These lines are being used as parents in breeding programs. Particularly, 2 lines that showed even yield at par with the best checks (Fig. 5C) are being tested in advance yield trials.

### Wheat Pre-Breeding for Impact

Conventional way to utilize germplasm bank accessions is to identify useful trait donors and then use them in pre-breeding. This approach is successful to improve specific trait and are being used in most breeding programs. However, in this investigation, exotics alleles were first brought into elite backgrounds and then useful alleles were selected. We pursued a three-way cross strategy (exotic/elite_1_//elite_2_) to generate PBLs, in a way so that each PBL possessed approximately 25% of the exotic and 75% of the elite genomes at an early stage. Therefore, exotic alleles were incorporated into elite backgrounds even before their trait values were identified. This strategy enabled investigation of greater number of genetic variants at a time and also allowed recombination between exotic and elite genomes to be exploited for genetic improvement of elites. Further, bulk selection in subsequent segregating generations (TC_1_F_2_ to TC_1_F_5_) helped in capturing maximum useful diversity. In addition, HB analysis suggests that this strategy retained rare and useful allelic variation. The simultaneous evaluation of the derived germplasm at multiple locations ensured minimum loss of useful diversity, identify useful, and novel diversity into well-adapted regional elite cultivars. The agronomic competitiveness of many PBLs with elite check lines further indicates that this approach addressed the bottlenecks of undesirable drag, possibly by breakage of haplotype blocks and thereby selection of useful alleles or interaction of exotic with elite alleles.

The ‘Seeds of Discovery’ project has used more than 1,000 exotic accessions to develop PBLs that have entered product pipelines in several breeding programs in India, Pakistan, UK, Brazil, Kenya, Australia, Canada Turkey, and Mexico (Supplementary Fig. 5C). A wheat pre-breeding pipeline (Supplementary Fig. 7) has been established in which these germplasm materials are being shared with researchers across the world in the form of the International Wheat Pre-breeding Nurseries (IWPN). Three IWPNs have been distributed and forthcoming will be available in coming years.

## Conclusion

Numerous publications have emphasized the importance of pre-breeding and gene bank use but seldom have made an effort practically. The present research elaborates the exhaustive efforts taken by SeeD (starting with 400,000 initial segregating pre-breeding lines) to bring in the untapped diversity of wheat exotics in gene bank into PBLs. CIMMYT provided first research breakthrough by providing semi-dwarf materials to the global research community. Followed by this, second generation germplasms were provided by CIMMYT i.e. synthetics. This is the third major impact-oriented germplasm infusion effort that has resulted in strategic development of large-scale pre-breeding germplasm (Fig. 1) followed by its deployment through breeding programs. Further, the use of high-density genotyping, in combination with multi-location phenotyping for a set of agronomic and stress tolerance traits, enabled the quantification of significant and positive genomic contributions from exotic wheat germplasm to progenies of their three-way crosses with pairs of elite wheat lines. The three-way crosses, followed by mild selection for essential agronomic traits during generation advancement, effectively captured many minor haplotypes from exotic germplasm bank accessions in the resultant progeny PBLs. The donors identified for heat, drought, and disease resistances, and for enhanced grain zinc concentration (up to 58 ppm), along with rare exotic haplotypes associated with the traits, demonstrated the important role that exotic germplasm can play in improvement of wheat elite lines globally. Under the worsening climate change scenarios and the anticipated threats of new emerging diseases for instance, wheat blast emergence in Bangladesh, the bridging germplasm created here can serve as a handy germplasm for both screening resistance donors and identification of candidate genes.

## Materials and Methods

Twenty-five elite CIMMYT wheat lines were used for developing pre-breeding germplasm. These 25 elites were either released varieties (Supplementary Table 10A) or performed well in multi-location evaluation trials. These genotypes have moderate to high levels of resistance to leaf and yellow rust and are widely adapted to different environmental conditions (personal communication, Ravi Singh CIMMYT).

Around 1,711 exotic accessions from CIMMYT’s germplasm bank, including 893 synthetic wheats (developed at CIMMYT by crossing durum wheat (*T*. *turgidum* subsp. *durum*) or emmer wheat (*T*. *dicoccum*) with diverse *Ae. tauschii* accessions), 784 landraces and 34 other materials, were used to make three-way crosses (exotic/elite_1_//elite_2_), resulting in 1,200 TC_1_F_1_ (top cross) populations. Only 244 of these TC_1_F_2_ populations were advanced, based on their agronomic performance. The diversity analysis of the exotics involved in generating these 244 populations revealed them genetically diverse (Supplementary Fig. 8). The exotic parents of the 244 TC_1_F_2_ populations were 125 CIMMYT synthetics: 50 accessions obtained from the International Center for Agriculture Research in Dry Areas (ICARDA), identified using the focused identification of germplasm strategy approach^29^; 33 heat-adapted materials from the Australian germplasm bank in Horsham, Victoria and termed ‘Australia hot’^15^; and 15 Iranian landraces, 13 Mexican landraces and 8 inbred lines from CIMMYT’s germplasm bank. The selected exotic accessions were grown along with staggered planting of the 25 elite lines to make F_1_ crosses that were subsequently crossed with a second elite line to form three-way cross populations.

The 244 three-way cross populations were advanced by selected bulk method up to TC_1_F_5_ stage. 8,157 TC_1_F_5_ plants were evaluated for plant type and disease performance of which 984 TC_1_F_5:6_ lines were selected. This process resulted in the elimination of all lines from 62 of the 244 populations, mainly based on poor agronomic performance and susceptibility to yellow rust. The selected 984 lines thus originated from 183 populations involving 165 exotic accessions. These 165 exotics included 75 landraces (23 ‘Australia hot’, 9 Iranian landraces, 10 Mexican landraces and 33 landraces from focused identification of germplasm strategy-FIGS), 86 synthetics and 4 other germplasm bank lines. The 165 exotics represented different gene pools and from parts of the world. The exotics and PBLs have been enlisted in Supplementary Table 10B and C.

### Development of Advanced Pre-Breeding Lines (PBLs)

The 244 TC_1_F_1_ plants were advanced to the TC_1_F_2_ generation and approximately 2000 seeds for each TC_1_F_2_ were grown in 50 m plots at CIMMYT’s experimental station at Ciudad Obregon, Mexico under drought- and heat-stress environments. Approximately 488,000 TC_1_F_3_ plants were visually selected for good performance, and spikes from them were selected and bulked for each cross. The TC_1_F_3_ bulks were grown at El Batan and Toluca, Mexico, for bulk advancement following mild selection under natural infection of yellow rust disease (with susceptible checks ‘Avocet’ and ‘Morocco’). The TC_1_F_4_ bulk populations were grown at the Ciudad Obregon station under managed heat- and drought-stress for a second round of selection, resulting in 8157 TC_1_F_4:5_ selections that were grown in 1-m^2^ plots for evaluations of plant-type (at El Batan) and resistance to yellow rust (at Toluca). 984 TC_1_F_5:6_ plants were selected and subsequently advanced as “pre-breeding lines” (PBLs). The 984 PBLs originated from 183 top-crosses that used 165 exotic accessions. The general pre-breeding strategy is outlined in Fig. 1.

### Genotypic Characterization

Genomic DNA was extracted from leaf samples harvested from each TC_1_F_5_ plant following a modified CTAB (cetyltrimethylammonium bromide) method^30^. DNA samples were quantified with a Nano-Drop 8000 spectrophotometer V 2.1.0. Genotypic characterization used DArTseq™ technology **(**http://www.diversityarrays.com/dart-application-dartseq**)** at the Genetic Analysis Service for Agriculture (SAGA) service unit at CIMMYT headquarters (Texcoco, Mexico). The methodology described by Vikram *et al*.^16^ was followed to generate a total of 58,378 high quality SNP markers. The main parameters to select markers were call rate (the proportion of samples with genotypic score and not recorded as missing data) and average reproducibility (the proportion of technical replicate assay pairs for which the marker score was consistent). 12,071 SNP markers belonging to 10,111 sequence tags were identified based on these criteria, out of which 7,180 were used for the final analysis (Supplementary Table 11). Chromosome location, marker order and genetic distances were defined based on a 64,000-marker DArT-seq consensus map released by Diversity Arrays Technology Pty Ltd. (DArT) **(**http://www.diversityarrays.com/sequence-maps**)**.

### Haplotype Characterization

Haplotypes were generated in R (http://www.R-project.org)^31^ using a script based on the algorithm from Gabriel *et al*.^32^. Briefly, 95% confidence bounds on D prime were generated and each comparison was called “strong LD”, “inconclusive” or “strong recombination”. A block was created if 95% of informative (i.e. non-inconclusive) comparisons were “strong LD”. This method defined pairs to be in strong LD if the one-sided upper 95% confidence bound on *D*’ was >0.98 and the lower bound above 0.7. The Hardy Weinberg p-value cut off was set to 0.001, and minimum marker allele frequency was set to 0.05. Individuals with more than 75% of missing data were excluded from haplotype construction. When multiple SNPs had the same genetic position, only the first marker was used in haplotype construction. The haplotypes were displayed as blocks of marker numbers and alleles. These were named with the prefix ‘HB’ for haplotype block, followed by a number for the chromosome [(1, 2, 3… until 21 (1 being 1A, 2 being 1B, 3 being 1D, etc. to 21 for 7D)], followed by a dot and incrementing numbers (1 to N, N being the total number of haplotypes) of the haplotype blocks along the chromosome. For example, *HB1.1* and *HB2.2* designate the first and second haplotypes on chromosomes 1A and 1B, respectively.

### Exotic Allele Contribution in Selected Crosses

To estimate the contribution of exotic accessions to derived PBLs, we analyzed ten crosses, each with ≥16 derived PBLs (Supplementary Table 2). Genetic composition of the PBLs was analyzed relative to the elite and exotic parents used in the crosses. The ABH script of Tassel^33^ was used to identify the genotypes homozygous for all the parents and for which the alleles differed between the elite and exotic parents. If any parent of the cross was heterozygous for a marker, then that marker was classified as of unidentified origin in the PBL. Markers for which all parents were homozygous, and for which exotic and elite parent alleles differed, were designated as follows in the PBLs: recombinants denoted by H when the marker was heterozygous; allele of elite origin as “A”, and of exotic origin as “B”. All markers originally without information were considered as missing. This was done for each PBL in each specific cross.

### GWA Analysis for Marker-Trait Associations

Genome-wide association (GWA) analysis was conducted for two population panels: (1) the panel of 984 PBLs and (2) a subset of 134 PBLs selected from the initial set of 984 PBLs based on agronomic performance at multiple locations. The covariance matrix was derived by PCA using the PRCOMP function from the STATS package in R^31^. The kinship matrix was calculated using the R package GAPIT. GWAS analysis was conducted in Plink version 1.07^34^ executed in R. A mixed linear model (MLM) utilizing PCA as fixed and kinship matrix as random effect was used. The Bayesian Information Criterion (BIC) was used to select the appropriate number of principal components for each trait^35^.

Significant marker-trait associations (MTAs) were declared using a threshold *p*-value within the bottom 0.1 percentile of the distribution. This approach avoids risk of type II error and has been used in recent studies for wheat^36^. A threshold *p*-value of 0.001 and 0.0001 corresponded to the bottom 0.1 percentile of the distribution for GY and yield components and for disease resistance, respectively. Hence, a marker was declared significant if it showed (a) *p*-value above the threshold and (b) deviation of its *p*-value from the normal distribution curve in the quantile-quantile (QQ) plot.

### Evaluation for Grain Yield under Heat, Drought and Irrigated Conditions

Phenotypic evaluation of the 984 PBLs for GY under heat, drought and irrigated treatments was conducted at CIMMYT’s experimental station near Ciudad Obregon (27 20°N, 109 54°W, 38 m ASL), Mexico. 984 PBLs were evaluated for GY under heat stress in 2016 and 2017, as well as for GY under drought- and well-irrigated conditions in 2016. Further, a sub-set of 200 best-performing PBLs was selected from the 2016 trial for evaluation in 2017. The experimental designs were alpha-lattices with two or three replications, and plot size of 2.0 and 4.8 m^2^ in 2016 and 2017, respectively. Whereas irrigated and drought experiments were sown in the second fortnight of November, the heat stress trials were sown in early March to expose them to 35–40 °C temperatures during anthesis. Agronomic management was the same for all trials, except for irrigation. Approximately 600 mm of water was provided in the irrigated and the heat experiments, while the drought experiment received ~200 mm of total soil moisture during the crop season. The statistical analyses to estimate means and variances were performed using R software.

### Evaluation of Grain Zinc Concentration

Zinc concentration was measured for grain of the 984 PBLs grown in a well-irrigated experiment with 2 replications at Ciudad Obregon in 2016. The 50 PBLs with highest grain Zn concentration (in 2016) were evaluated again in 2017 in an experiment with two replications. Grain Zn concentration (µg g^−1^) was estimated using a “bench-top,” non-destructive, energy-dispersive X-ray fluorescence spectrometry (EDXRF) instrument (model X-Supreme 8000, Oxford Instruments plc, Abingdon, UK) according to the method described by Paltridge *et al*.^37^.

### Evaluation of Disease Resistances

Powdery Mildew (*Blumeria graminis* f. sp*. tritici – Bgt*) resistance of the 134 PBL sub-set was evaluated at Malan research station in Himachal Pradesh, India (31.1048°N, 77.1734°E), which is a natural hotspot for the disease. Experiments used randomized complete block designs (RCBD) with two replications of 1.0 m^2^ plots in which row-to-row spacing was 20 cm. Sowing was in the first fortnight of November (2016 and 2017) and standard agronomic practices (as explained in the above section) were followed. The susceptible varieties Lehmi and HPW 155 were sown between every tenth test genotype and on the outer boundaries of the plots for use as susceptible checks and to multiply and spread inoculum. The experiments were dust inoculated with a locally available isolate of *Bgt*. The inoculum was multiplied on seedlings of HPW155, Lehmi, and Agra Local grown in 4-inch pots. Data were recorded for overall disease reaction on a 0–9 scale as described by Saari and Prescott^38^.

The 134 PBLs were evaluated against yellow rust (*Puccinia striiformis* f. sp. *tritici*) virulent pathotypes 46S119 and 78S84, at IARI-New Delhi (30.90284 N, 75.79692 220 m ASL), and PAU, Ludhiana (30.90284 N, 75.79692 E 250 m ASL), India. Experiments were performed at both locations during two consecutive years, using RCBDs with two replications of 0.5 m^2^ plots with 20 cm inter-row spacing. Avocet was used as a susceptible check grown all-around the experimental blocks. Methodology explained by Hao *et al*.^39^. was used to inoculate spreader/border rows. Disease symptoms were scored when the susceptible check showed 100% yellow rust infection. The percent of infection was estimated according to the modified Cobb’s scale^40^.

### Bioinformatics Analysis of Significant Associations

Efforts were made to identify HB markers within the predicted gene coding sequence. Four HBs, i.e. HB16.10, HB18.1, HB10.5 and HB5.23, showing consistent significant effects (for GY-HB16.10, HB18.1, HB10.5 and yellow rust-HB5.23) in GWA using multi-location data, were subjected to bioinformatics analyses. Sixty base pair sequences of each clone ID associated with each SNP marker were anchored to the Refseq. 1 physical map of wheat using BLAST. Markers were anchored based on the top hit (taking into account both query length and percentage match). All genes were extracted between the outermost markers associated with each haplotype +/− 500 Kbp. The size of each interval and number of genes within these intervals are enlisted in Supplementary Table 12. Annotated high confidence genes within GY-associated haplotypes were then submitted to Knetminer^17^ to identify genes that have previously been implicated in determining GY for multiple plant species.

The world map presented in Supplementary Fig. 8 was constructed using ESRI ArcGIS Desktop software^41^.

## Electronic supplementary material

Harnessing genetic potential of wheat germplasm banks through impact-oriented-prebreeding for future food and nutritional security

Dataset 2

Dataset 3

Dataset 6

Dataset 9

Dataset 10

Dataset 11
