# Supplementary material for: Harnessing genetic potential of wheat germplasm banks through impact-oriented-prebreeding for future food and nutritional security
Source: Sci Rep. 2018 Aug 21;8:12527. doi: 10.1038/s41598-018-30667-4 (PMC6104032; doi:10.1038/s41598-018-30667-4)
Supplement: Supplementary file 1 — Harnessing genetic potential of wheat germplasm banks through impact-oriented-prebreeding for future food and nutritional security [file 41598_2018_30667_MOESM1_ESM.pdf]

Harnessing genetic potential of wheat germplasm banks through impact-oriented-prebreeding for future food and nutritional security

Sukhwinder Singh[1*ǂ], Prashant Vikram[1ǂ], Deepmala Sehgal[1ǂ], Juan Burgueño[1], Achla Sharma[2], Sanjay K. Singh[3], Carolina P. Sansaloni[1], Ryan Joynson[4], Thomas Brabbs[4], Cynthia Ortiz[1], Ernesto Solis-Moya[5], Velu Govindan[1], Naveen Gupta[6], Harminder S. Sidhu[6], Ashwani K. Basandrai[7], Daisy Basandrai[7], Lourdes Ledesma-Ramires[5], Maria P. Suaste-Franco[5], Guillermo Fuentes-Dávila[8], Javier I. Moreno[9], Kai Sonder[1], Vaibhav K. Singh[10], Sanjay Singh[11], Sajid Shokat[12,13], Mian A. R. Arif[12], Khalil A. Laghari[13], Puja Srivastava[2], Sridhar Bhavani[14], Satish Kumar[3], Dharam Pal[15], Jai P. Jaiswal[16], Uttam Kumar[6], Harinder K. Chaudhary[7], Jose Crossa[1], Thomas S. Payne[1], Muhammad Imtiaz[18], Virinder S. Sohu[2], Gyanendra P. Singh[3], Navtej S. Bains[2], Anthony Hall[4,19], Kevin V. Pixley[1*]

[1]International Maize and Wheat Improvement Center (CIMMYT), Carretera México-Veracruz Km. 45, El Batán, Texcoco, México, C.P. 56237
[2]Department Plant Breeding & Genetics, Punjab Agriculture University, Ludhiana-141004, India
[3]ICAR-Indian Institute of Wheat and Barley Research, Karnal-132001, India
[4]Earlham Institute, Norwich, Norfolk, NR4 7UG, UK
[5]Carretera Celaya-San Miguel de Allende, Km .6.5, C.P. 38110, Celaya, Guanajuato, México
[6]Borlaug Institute for South Asia (BISA), CIMMYT, Ladhowal, Punjab, 141004, India
[7]CSK Himachal Pradesh Agricultural University Palampur, H.P. India, 176062
[8]INIFAP-CIRNO, Campo Experimental Norman E. Borlaug, Apdo. Postal 155, Km 12 Norman E. Borlaug, Cd. Obregon, Sonora, Mexico CP 85000
[9]INIFAP, Interior Parque Los Colomos S/N, Colonia Providencia, CP. 44660, Guadalajara, Jalisco
[10]ICAR-Indian Agricultural Research Institute (IARI), New Delhi - 110 012, INDIA
[11]National Research Center for Plant Biotechnology, New Delhi - 110 012, INDIA
[12]Nuclear Institute for Agriculture and Biology, Faislabad -38000, Pakistan
[13]Department of Plant and Environmental Sciences, Crop Science, University of Copenhagen, Højbakkegård Allé 13, DK-2630, Taastrup, DENMARK
[14]Nuclear Institute of Agriculture, Tando Jam, Sindh, - 70050, Pakistan,
[15]CIMMYT - World Agroforestry Centre (ICRAF), United Nations Avenue, Gigiri. P.O. Box 1041–00621, Nairobi, Kenya
[16]ICAR-Indian Agricultural Research Institute, Regional Station, Shimla-171004, India
[17]Department of Genetics & Plant Breeding, G.B. Pant University of Agriculture & Technology, Pantnagar- 263145 Uttarakhand, India
[18]CIMMYT – Pakistan, NARC CSI Complex, Park Road, Islamabad- 44000, Pakistan
[19]School of Biological Sciences, University of East Anglia, Norwich Research Park, Norwich NR4 7TJ, UK

[ǂ] Equal contributions
[*]Corresponding authors:

Sukhwinder Singh | Wheat Lead- Seeds of Discovery
International Maize and Wheat Improvement Center (CIMMYT)
Tel: +52 (595) 952 1900 (2104) Email: suk.singh@cgiar.org
Kevin Pixley | Director, Genetic Resources Program
International Maize and Wheat Improvement Center (CIMMYT)
Carretera México-Veracruz Km. 45, El Batán, Texcoco, México, C.P. 56237
Tel: +52-595-9521900 (1397) Email: k.pixley@cgiar.org

Supplementary Table 1. Haplotype variation among three sets of germplasm; exotic, pre breeding, and elite

Chromosome	Germplasm set	Total number of haplotypes	Length of haplotypes (number of SNPs in a haplotype): min to max	Genomic regions in PBLs where large HBs in elites were broken (genetic position in cM based on 64K map)
1A	Elite	10	2-36	
	Exotic	33	2-7	
	PBLs	32	2-10	190.3-194.7, 195.8- 196.6, 204.3-204.7, 206.7-207.0, 208.1-218.3, 225.2-226.0, 226.4-228.4, 313.4-343.3
1B	Elite	4	2-3	
	Exotic	23	2-8	
	PBLs	14	2-5	
1D	Elite	1	4	
	Exotic	14	2-6	
	PBLs	11	2-3	75-78
2A	Elite	7	2-6	
	Exotic	19	2-3	
	PBLs	18	2-6	
2B	Elite	9	2-10	
	Exotic	27	2-5	
	PBLs	30	2-6	68-69
2D	Elite	3	3-8	
	Exotic	20	2-8	

	PBLs	10	2-12	114-118
3A	Elite	5	2-8	
	Exotic	16	2-5	
	PBLs	20	2-5	
3B	Elite	10	2-8	
	Exotic	23	2-5	
	PBLs	33	2-6	130.8-131.5, 185.4 – 185.6, 285.5 – 286.1
3D	Elite	1	5	
	Exotic	11	2-22	
	PBLs	9	2-5	
4A	Elite	4	3-7	
	Exotic	11	2-5	
	PBLs	14	2-4	181.7 – 190.0, 211.0-215.4
4B	Elite	4	2-9	
	Exotic	9	2-4	
	PBLs	6	2-3	
4D	Elite	1	3	
	Exotic	8	2	
	PBLs	6	2	
5A	Elite	10	2-5	
	Exotic	20	2-6	
	PBLs	20	2-5	
5B	Elite	11	2-15	
	Exotic	28	2-6	

	PBLs	37	2-4	23.3-23.8, 23.9-25.5, 63.7-69.1, 100.4-106.1, 107.2-115.7
5D	Elite	1	7	
	Exotic	6	2-3	
	PBLs	3	2-5	217.9-228.8
6A	Elite	5	3-8	
	Exotic	13	2-4	
	PBLs	19	2-6	110.8-112.3
6B	Elite	3	2-6	
	Exotic	14	2-4	
	PBLs	15	2-6	
6D	Elite	4	2-3	
	Exotic	10	2-39	
	PBLs	6	2-3	
7A	Elite	11	2-7	
	Exotic	25	2-6	
	PBLs	28	2-6	
7B	Elite	10	2-8	
	Exotic	16	2-3	
	PBLs	23	2-5	61.6-75.1, 76.7-79.0
7D	Elite	1	2	
	Exotic	21	2-23	
	PBLs	6	2-4	

Supplementary Table 2. Contribution of exotic and elite alleles in ten crosses with equal to or more than 16 pre-breeding lines selected after 7 cycles of selection (separate excel sheet attached)

Supplementary Table 3. Haplotype associations with grain yield and related traits (separate excel sheet attached)

Supplementary Table 4. Haplotype blocks associated with grain yield under heat stress and irrigated situation

Haplotype	Chromosome	Irrigated		Heat-2016		Heat-2017	
		P-value	R^2 (%)	*P*-value	R^2 (%)	*P*-value	R^2 (%)
HB18.1	6D	9.88E-05	2.8	3.30E-12	6.1	5.3076E-07	3.7
HB8.22	3B	8.33E-04	3.4	6.29E-04	3.6	0.00204	3.0
HB16.10	6A	3.42E-05	4.2	9.90E-04	3.4	0.00045675	3.4
HB18.2	6D	1.38E-06	3.7	3.30E-12	6.2	2.44E-04	3.0

Supplementary Table 5A. Haplotype associations for yellow rust resistance in 984 PBLs

Haplotype	Chromosome	*P*-value	Location-PAU	Location-Palampur
HB1.21	1A	0.00103		
HB1.31	1A	0.000113		**
HB1.32	1A	8.16E-05		**
HB2.1	1B	0.000924		***
HB2.7	1B	3.76E-06	**	**
HB3.4	1D	0.000426	***	
HB3.7	1D	0.000151		**
HB5.2	2B	0.00155		***
HB5.23	2B	0.00138	**	**
HB19.4	7A	1.74E-05		**
HB20.22	7B	0.00309	**	**

P-Value significance level: *: < 0.01; **: < 0.01; *** :<0.0001

Supplementary Table 5B. Haplotype associations for powdery mildew resistance in the pre-breeding germplasm set

Haplotype	Chromosome	*P*-value	R^2
HB14.36	5B	3.17E-05	0.0308
HB17.11	6B	1.09E-04	0.0281

Supplementary Table 6. Genes found within trait-associated intervals along with their molecular function and genome annotation (separate excel sheet attached)

Supplementary Table 7. Eight allelic variants of gene Traes_6DS_84A4D85F.1 with SIFT score <0.05

Variant ID	Chromosome: bp	Alleles	Class	Source	Conseq.Type	SIFT	Transcript
EPITAEV07251078	6D:3099529	G/C	SNP	Inter-homoeologous	Missense variant	0	Traes_6DS_84A4FD85F1
EPITAEV01106843	6D:3099718	T/G	SNP	Inter-homoeologous	Missense variant	0	Traes_6DS_84A4FD85F1
EPITAEV01106544	6D:3099856	T/C	SNP	Inter-homoeologous	Missense variant	0	Traes_6DS_84A4FD85F1
EPITAEV01106553	6D:3099833	G/A	SNP	Inter-homoeologous	Missense variant	0.01	Traes_6DS_84A4FD85F1
EPITAEV01106611	6D:3099439	C/T	SNP	Inter-homoeologous	Missense variant	0.02	Traes_6DS_84A4FD85F1
EPITAEV01106815	6D:3099471	G/T	SNP	Inter-homoeologous	Missense variant	0.02	Traes_6DS_84A4FD85F1
EPITAEV01106694	6D:3099160	T/C	SNP	Inter-homoeologous	Missense variant	0.03	Traes_6DS_84A4FD85F1
EPITAEV01106840	6D:3099708-3099709	GG/CA	substitution	Inter-homoeologous	Missense variant	0.04	Traes_6DS_84A4FD85F2

Supplementary Table 8A. Pre-breeding lines identified as potential donors for grain yield under heat stress

GID	Exotic parent type	2015-16	2016-17
		------ kg ha^{-1} ------	
7641495	Synthetic	2261	2346
7644075	Synthetic	2325*	2418*
7645422	Synthetic	2338*	2488*
7645970	Synthetic	2214	2407*
7689940	Landrace	2415*	2362
BAJ #1	Check	2144	2216
VOROBEY	Check	1769	1985
SOKOLL	Check	NA	2023
LSD(0.05)		526	386

In crop season 2015-16 and 2016-17, 984 and 230 PBLs respectively, were evaluated under heat stress environment at Ciudad Obregon, Mexico. The number of PBLs with grain yield equal/comparable to Baj#1 and Vorobey (checks) in 2015-16 were **610** and **908** respectively, and, in 2016-17 they were **165** and **193** respectively. *: PBLs yielding significantly higher than Vorobey.

Supplementary Table 8B. Pre-breeding lines performing comparable or better than checks for grain yield under drought stress

GID	Exotic parent type	2015-16	2016-17
		------- kg ha^{-1} -------	
7643084	Synthetic	3587	4510
7642492	Synthetic	3480	4574
7688508	Landrace	3360	4787*
7687479	Synthetic	3167	5198*
7642491	Synthetic	2766	5151*µ
VOROBEY	Check	3346	4613
BAJ #1	Check	3111	4858
SOKOLL	Check	NA	3968
LSD(0.05)		868	457

A total of **984** and **230** PBLs were evaluated in crop season of 2015-16 and 2016-17 respectively, under drought stress environment at Ciudad Obregon, Mexico. Of these, grain yield of **940** and **799** PBLs were equal/comparable to Baj#1 and Vorobey, in the crop season 2015-16 respectively. In the 2016-17 season, PBLs with grain yield equal/ comparable to Baj#1 and Vorobey were **43** and **89,** respectively. ***: Three** PBLs yielded significantly higher than Vorobey in the crop season 2016-17; **µ: One** PBL yielded significantly higher than Baj#1 in the crop season 2016-17.

Supplementary Table 9. List of exotics with minor haplotypes associated with agronomic traits

Supplementary Table 10. List of elites, exotics and pre-breeding lines used in this study. Separate excel sheet attached.

10-A: Salient features of the 25 elites that were either released varieties or better performing lines in CIMMYT's multi-location trials. These elites were used for developing pre-breeding germplasm

populations; 10-B: Details of 165 exotics involved in 183 crosses representing 984 pre-breeding lines; 10-C: Details of 183 three-way crosses representing 984 pre-breeding lines

Supplementary Table 11. (**A) Details of 10,111 SNP makers used for analysis in this study; (B) Details of 7,180 SNP markers used for haplotype analysis in this study. Separate excel sheet attached**

Supplementary Table 12. Location and size of QTL intervals and number of annotated genes

Interval	Chromosome	Interval start	Interval end	Interval size (Mbp)	Number of Genes	Number of GY associated genes*
HB5.23	2B	657986441	690277442	32.29	279	(NB-LRR Resistance Associated genes)10
HB10.5	4A	123726509	137610108	13.88	61	(GY related genes) 4
HB16.10	6A	418039034	446592270	28.55	138	(GY related genes) 14
HB18.1	6D	4555134	6843670	2.29	48	(GY related genes) 6

*Flagged as previously associated with grain yield by KnetMiner

SUPPLEMENTARY FIGURE LEGENDS

Fig. S1. Pictorial presentation of haplotype blocks comparison between of elite and PBL haplotype block map. Figure shows the distribution of same 36 SNPs in elite and PBL haplotype map

Fig. S2. SNP allele profiles on all 21 chromosomes (columns) for pre-breeding lines (PBLs) of 10 crosses (rows). Black lines delimit chromosomes and crosses. Black dots inside the black lines are missing values, while blue and orange dots represent elite- and exotic-specific alleles, respectively. Green color corresponds to unidentified, or alleles originating from recombination of elite and exotics. Chromosomal regions where elite, exotic or recombinant alleles prevailed in the PBLs appear as vertical patterns of the same color.

Fig. S3: Phylogenetic tree of the *Traes_6DS_84A4D85F.1* candidate gene, from EnsemblPlants database, (http://plants.ensembl.org/index.html) showing closest similarity with the gene *F775_22033* in *Aegilops tauschii*, coding for isoflavone reductase IRL-like protein.

Fig. S4: BLAST analysis of two SNPs in HB18.1 revealed a significant hit for the SNP 2 (clone ID 1067078), with a candidate gene, *Traes_6DS_84A4D85F.1*. This gene is homologous to the rice gene *LOC_Os06g27770.1*, coding for isoflavone reductase. Allelic variants are shown in UTR, coding and intron regions of the gene (http://plants.ensembl.org/index.html). The dark yellow colored rectangular boxes represent missense mutations (causing deleterious amino acid changes) in the coding region.

Fig. S5. Average TKW of PBLs with alleles AC, AT and TC (X-axis) for HB18.2. PBLs with haplotype AC had highest TKW. The haplotype AC was present in 6% of PBLs.

Fig. S6. Different regions in the world map (i) to which the exotic parents belong and (ii) where pre-breeding germplasms have been shared

Fig. S7. Presenting number of crosses made in the last 5 years for wheat pre-breeding and development stages of germplasm and anticipated product delivery timeline has been illustrated

Fig. S8. Graph showing a three dimensional principal component analysis graph of 1711 gene bank accessions out of which 244 exotics in different genetic spaces. The selected and unselected genotypes are presented with red and yellow dots respectively

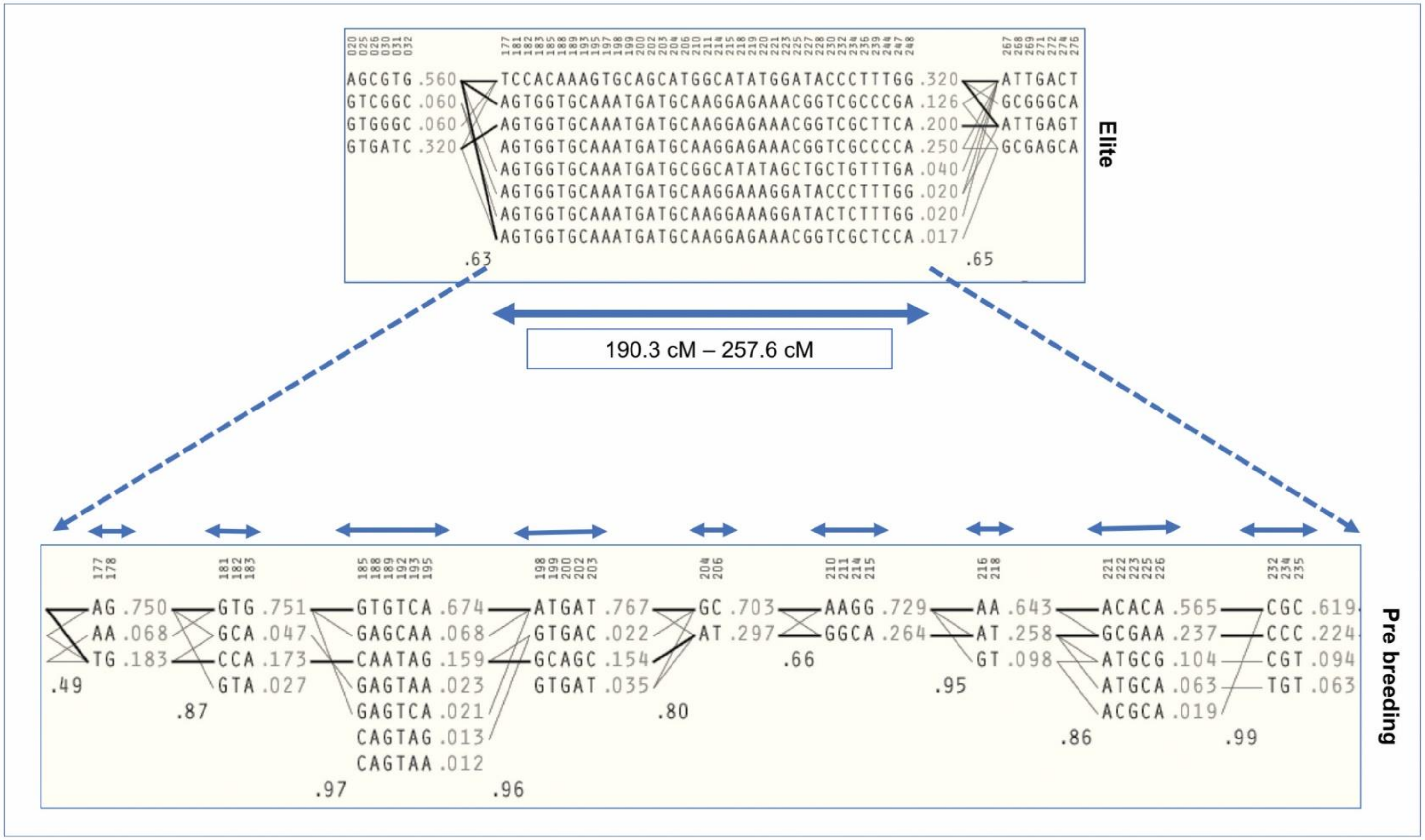
Elite
AGCGTG .560
GTCGGC .060
GTGGGC .060
GTGATC .320
TCCACAAAGTGCAGCATGGCATATGGATACCCTTTGG .320
AGTGGTGCAAATGATGCAAGGAGAAACGGTCGCCCGA .126
AGTGGTGCAAATGATGCAAGGAGAAACGGTCGCTTCA .200
AGTGGTGCAAATGATGCAAGGAGAAACGGTCGCCCCA .250
AGTGGTGCAAATGATGCGGCATATAGCTGCTGTTTGA .040
AGTGGTGCAAATGATGCAAGGAAAGGATACCCTTTGG .020
AGTGGTGCAAATGATGCAAGGAAAGGATACTCTTTGG .020
AGTGGTGCAAATGATGCAAGGAGAAACGGTCGCTCCA .017
ATTGACT
GCGGGCA
ATTGAGT
GCGAGCA
.63
.65
190.3 cM – 257.6 cM
Pre breeding
AG .750
AA .068
TG .183
.49
GTG .751
GCA .047
CCA .173
GTA .027
.87
GTGTCA .674
GAGCAA .068
CAATAG .159
GAGTAA .023
GAGTCA .021
CAGTAG .013
CAGTAA .012
.97
ATGAT .767
GTGAC .022
GCAGC .154
GTGAT .035
.96
GC .703
AT .297
.80
AAGG .729
GGCA .264
.66
AA .643
AT .258
GT .098
.95
ACACA .565
GCGAA .237
ATGCG .104
ATGCA .063
ACGCA .019
.86
CGC .619
CCC .224
CGT .094
TGT .063
.99

Fig.S1

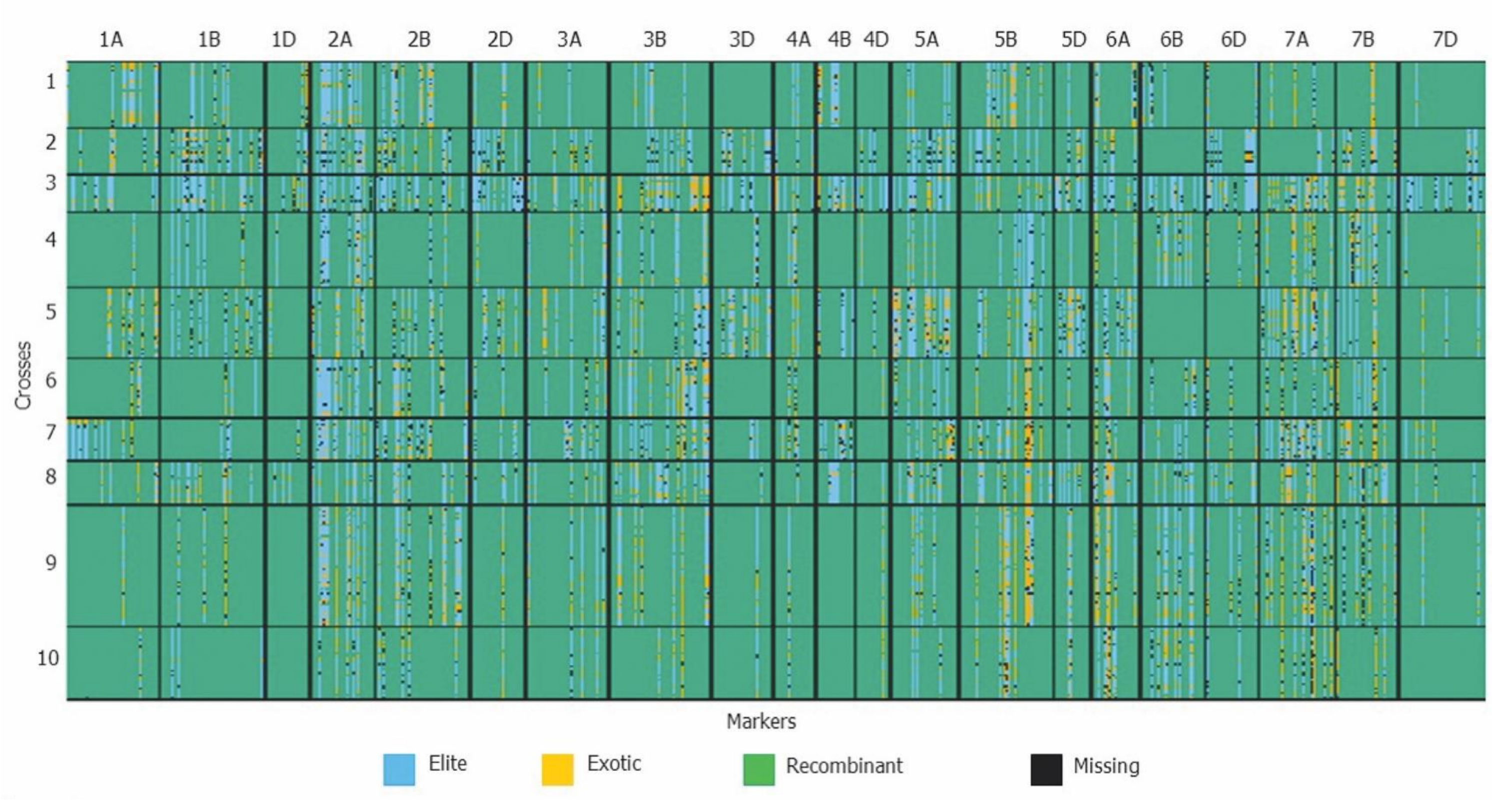
1A
1B
1D
2A
2B
2D
3A
3B
3D
4A
4B
4D
5A
5B
5D
6A
6B
6D
7A
7B
7D
Crosses
1
2
3
4
5
6
7
8
9
10
Markers
Elite
Exotic
Recombinant
Missing

Fig.S2

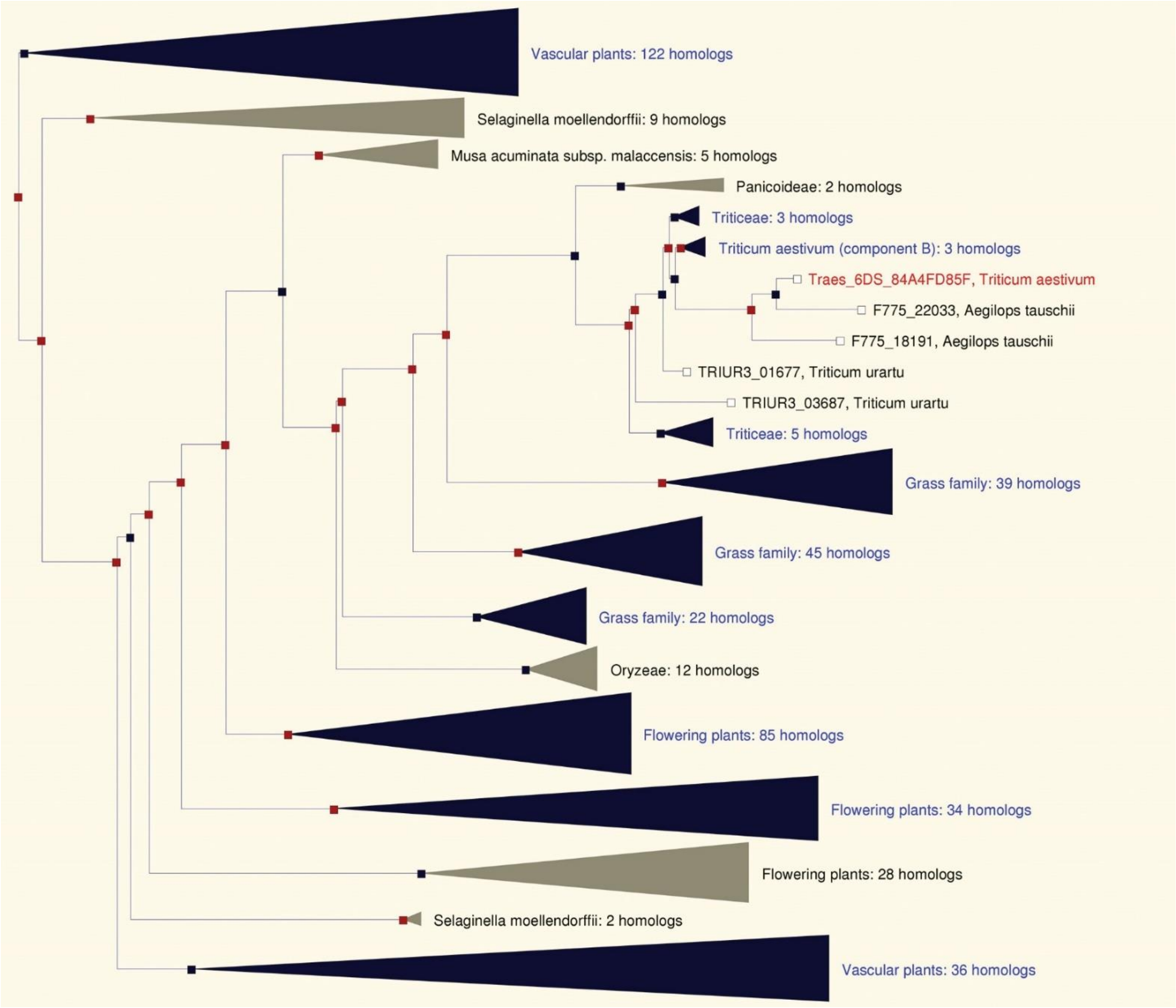

Vascular plants: 122 homologs
Selaginella moellendorffii: 9 homologs
Musa acuminata subsp. malaccensis: 5 homologs
Panicoideae: 2 homologs
Triticeae: 3 homologs
Triticum aestivum (component B): 3 homologs
Traes_6DS_84A4FD85F, Triticum aestivum
F775_22033, Aegilops tauschii
F775_18191, Aegilops tauschii
TRIUR3_01677, Triticum urartu
TRIUR3_03687, Triticum urartu
Triticeae: 5 homologs
Grass family: 39 homologs
Grass family: 45 homologs
Grass family: 22 homologs
Oryzeae: 12 homologs
Flowering plants: 85 homologs
Flowering plants: 34 homologs
Flowering plants: 28 homologs
Selaginella moellendorffii: 2 homologs
Vascular plants: 36 homologs

Fig.S3

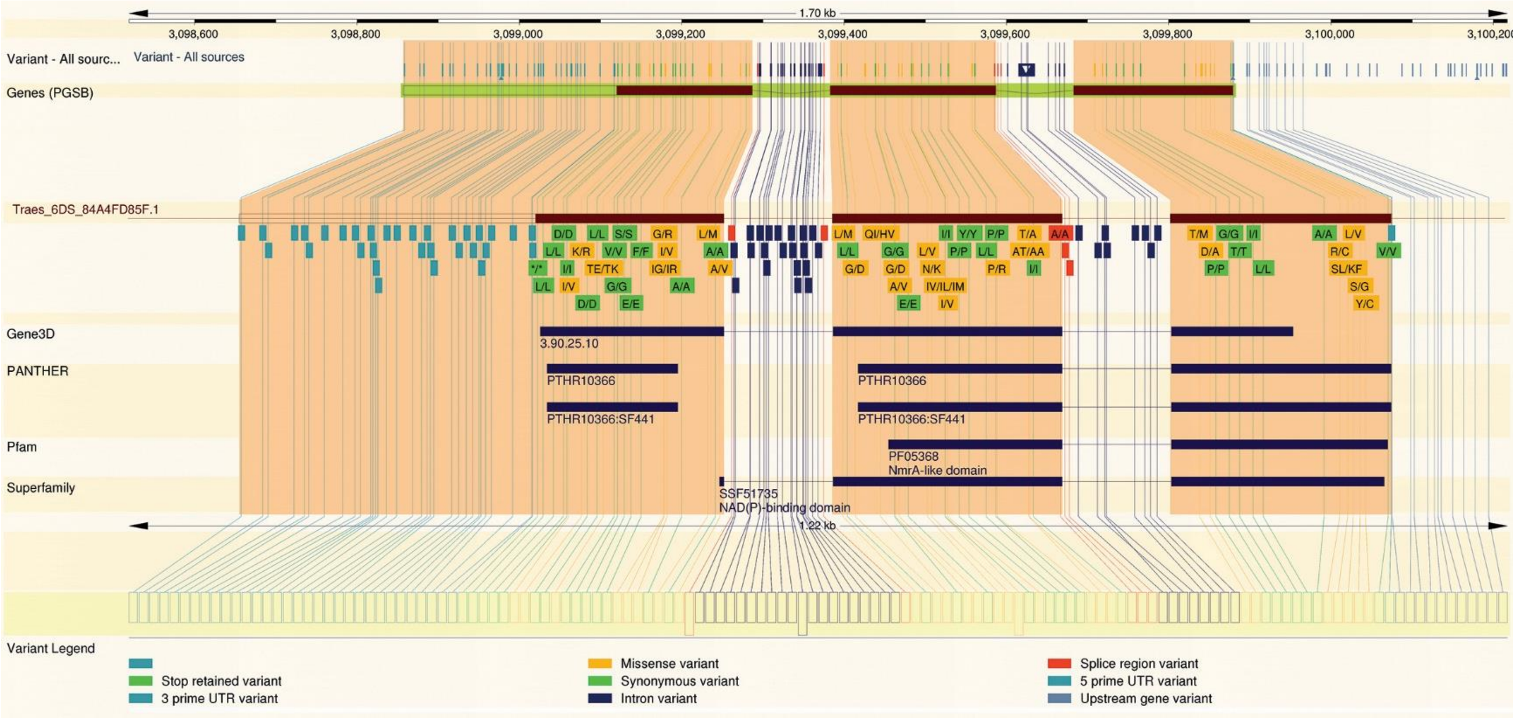
1.70 kb
3,098,600
3,098,800
3,099,000
3,099,200
3,099,400
3,099,600
3,099,800
3,100,000
Variant - All sourc...
Variant - All sources
Genes (PGSB)
Traes_6DS_84A4FD85F.1
Gene3D
3.90.25.10
PANTHER
PTHR10366
PTHR10366:SF441
Pfam
PF05368
NmrA-like domain
Superfamily
SSF51735
NAD(P)-binding domain
1.22 kb
Variant Legend
Stop retained variant
3 prime UTR variant
Missense variant
Synonymous variant
Intron variant
Splice region variant
5 prime UTR variant
Upstream gene variant

Fig.S4

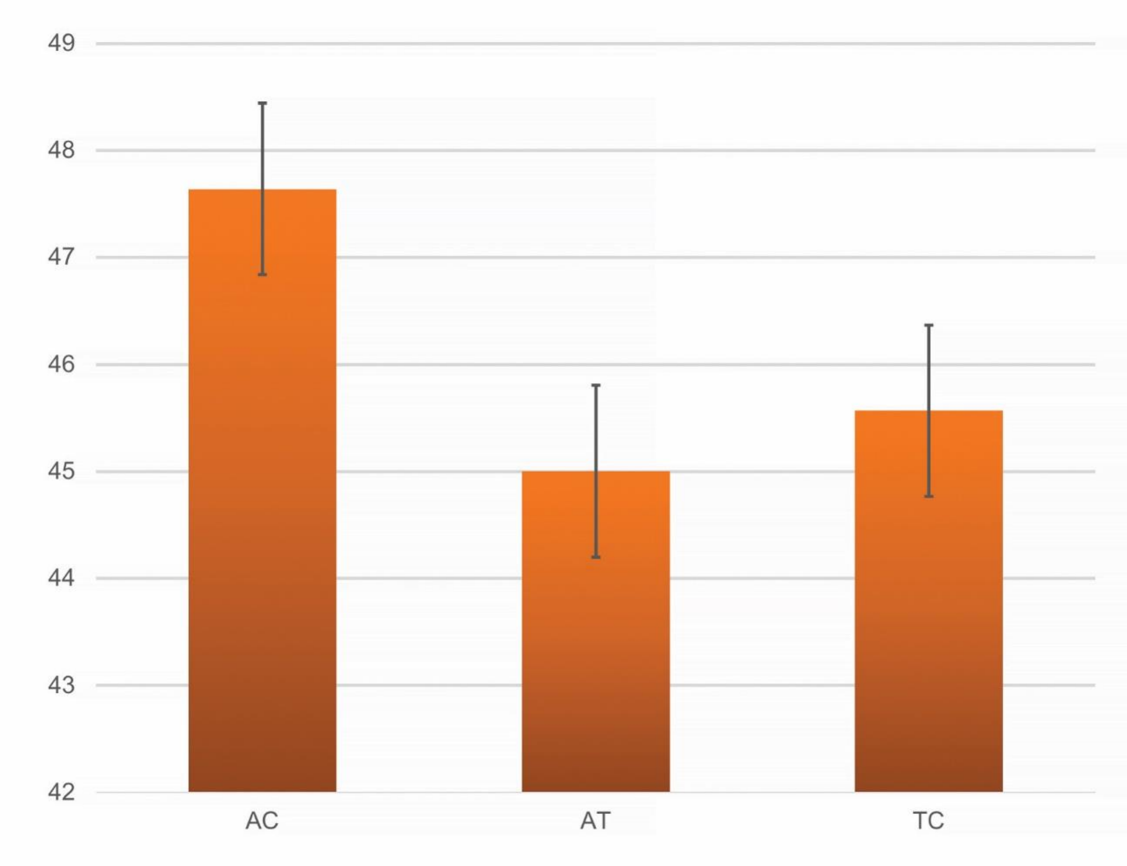

Fig.S5

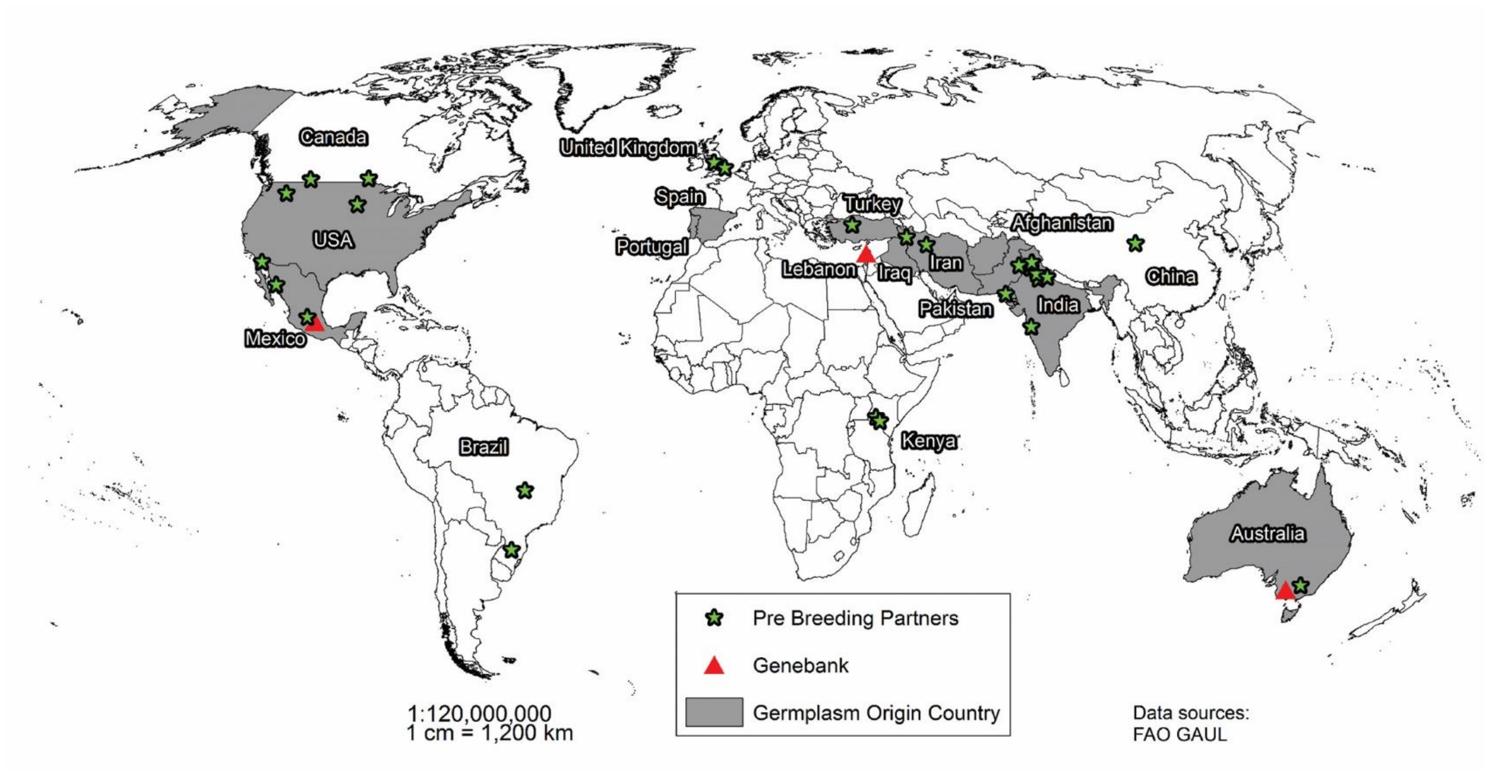
Canada
United Kingdom
Spain
Portugal
Turkey
Afghanistan
USA
Lebanon
Iraq
Iran
China
Pakistan
India
Mexico
Brazil
Kenya
Australia
Pre Breeding Partners
Genebank
Germplasm Origin Country
1:120,000,000
1 cm = 1,200 km
Data sources:
FAO GAUL

Fig.S6

2016	200	2021	TC_1F_3
2015	500	2020	TC_1F_4
2014	200	2019	TC_1F_5
2013	126	2018	TC_1F_6
**2012**	**244**	**2017**	$\mathbf{TC_1F_7}$
Crossing Initiated	Number of crosses	Mobilization to breeding pipelines	Filial stage of pre breeding product

Fig.S7

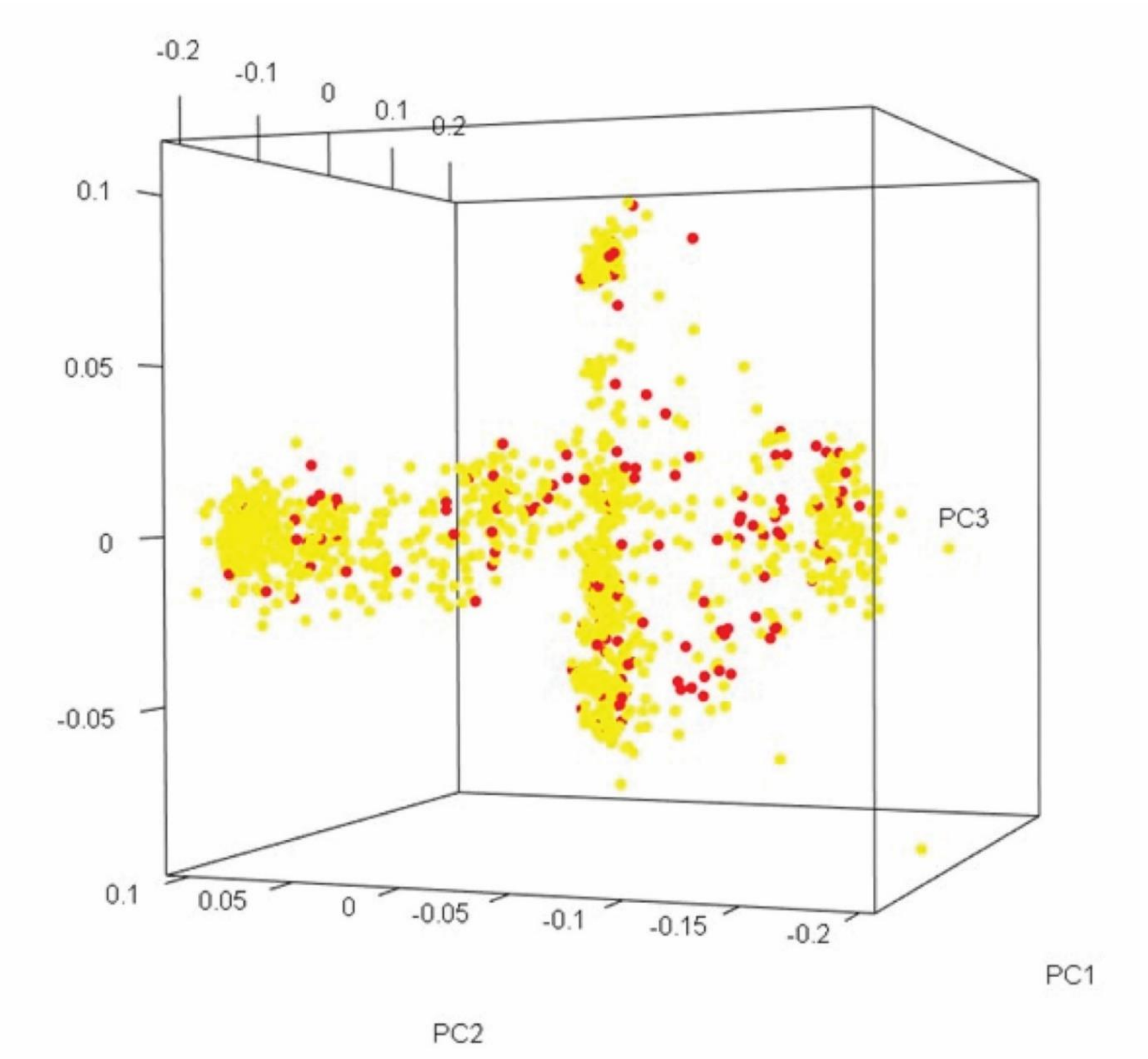

Fig.S8
